# Silencing CAPN2 Expression Inhibited Castration-Resistant Prostate Cancer Cells Proliferation and Invasion via AKT/mTOR Signal Pathway

**DOI:** 10.1155/2017/2593674

**Published:** 2017-02-09

**Authors:** Pu Li, Chenkui Miao, Chao Liang, Pengfei Shao, Zengjun Wang, Jie Li

**Affiliations:** State Key Laboratory of Reproductive Medicine and Department of Urology, The First Affiliated Hospital, Nanjing Medical University, Nanjing 210029, China

## Abstract

The mRNA expression of CAPN2 was upregulated in CRPC cells (DU145 and PC3) than that in non-CRPC cells. Silencing CAPN2 expression could inhibit DU145 and PC3 cells proliferation by cell cycle arrest at G1 phase. Knockdown of CPAN2 level suppressed the migration and invasion capacity of CRPC cells by reducing matrix metalloproteinase-2 (MMP-2) and MMP-9 activation, as well as repressing the phosphorylation protein expression of AKT and mTOR. In addition, we found that the expression of CAPN2 was elevated in Pca tissues than that in normal control tissues. Therefore, we showed the important roles of CAPN2 in the development and progression in CRPC cells, suggesting a new therapeutic intervention for treating castration-resistant prostate cancer patients.

## 1. Introduction

Castration-resistant prostate cancer (CRPC) is one of the most commonly diagnosed human cancers in men and the second cause of cancer-related death for Western men [1]. Recently, its incidence and mortality have been continuously rising among Chinese men [2]. As the progress of the disease, invasion and metastasis are the main characteristics of malignant tumor. In spite of current available treatments, CRPC becomes metastasis and castration-resistant and advanced metastatic CRPC may be incurable [3]. Therefore, a novel progressive and prognostic CRPC maker providing valuable information for patients is urgently needed. Cancer metastasis was a multistep process, including invasion and metastasis in tumor cells, alterations of the tumor microenvironment, and degradation of the extracellular matrix (ECM). And degradation of the ECM was considered to play a crucial role in the formation of tumor metastasis [4]. Matrix metalloproteinases (MMPs) such as MMP-2 and MMP-9 contribute to ECM degradation leading to tumor invasion and metastasis.

The calpain system was a big family of calcium-activated protease, which was known to play important roles in cell proliferation, migration, and invasion [5]. CAPN2, also known as m-calpain, was a member of the calpains family. Currently, the two special isoforms *μ*-calpain (CAPN1) and m-calpain (CAPN2) have been fully studied [6]. And in 1996, Shiba et al. first reported the relationship between the calpains and cancer [7]. More recent evidences have showed the important role of CAPN2 in both carcinogenesis and tumor progression [8–11]. The high expression of CAPN2 in basal-like or triple-negative breast cancer was significantly associated with clinical outcome of patients and was conformed in an independent cohort of patients [8]. In addition, knockdown of CAPN2 mRNA expression reduced breast cancer cell invasion by regulating invadopodia dynamics [12]. Decreased CAPN2 expression using shRNA or chemical inhibition activity reduced glioblastoma cell invasion by 90% [11]. Moreover, silencing CAPN2 inhibited the migrative and invasive potentials of hepatocellular carcinoma cells by attenuating the MMPs secretion [10]. Furthermore, CAPN2 expression was also determined in CRPC samples, and its mRNA expression was significantly increased in metastatic prostate cancer compared with normal prostate by cDNA microarray [13, 14]. These results strongly demonstrated that CAPN2 may aim to reduce tumor metastasis and act as a potential treatment for special cancers.

Although the mechanism of calpain in tumor progression was well elucidated, how CAPN2 regulates CRPC cell migration and invasion was little known. In this study, we used qRT-PCR to determine CAPN2 levels in CRPC cell lines. Using siRNA based knockdown of CAPN2, we demonstrate that CAPN2 expression was required for CRPC cell migration and invasion through reducing MMP-2 and MMP-9 levels. Furthermore, silencing CAPN2 expression inhibiting CRPC cell proliferation was determined by flow cytometry. Furthermore, we used qRT-PCR, Western blot, and immunohistochemistry analysis to investigate the CAPN2 levels in clinical Pca tissues. Our results suggest that CAPN2 plays important roles in the invasive and metastatic potential of CRPC cells and may act as a candidate target for human CRPC diagnosis and therapy.

## 2. Materials and Methods

### 2.1. Clinical Samples and Cell Culture

The tissues and nonmalignant prostate tissues were obtained from patients who underwent radical prostatectomy at First Affiliated Hospital of Nanjing Medical University, China. All the samples after surgery were immediately frozen in liquid nitrogen and stored at –80°C until further analysis. Only samples containing >70% tumor cells were used for the extraction of total RNA. The use of clinical samples was approved by the medical ethics committee of our hospital (Table 1). The human prostate cancer cell lines (22RV1, LNCaP, DU145, and PC3) were purchased from the Cell Bank Type Culture Collection of the Chinese Academy of Sciences (Shanghai, China). The CRPC cell lines DU145 and PC3 human prostatic carcinoma cell lines were cultured in F-12K Nutrient Mixture (Gibco, USA), and 22RV1 and LNCaP were cultured in RPMI-1640 (Gibco, USA), all supplemented with medium containing 10% fetal bovine serum (FBS, Gibco, USA) and at humidified atmosphere containing 5% CO_2_ at 37°C.

### 2.2. RNA Isolation and qPCR

According to the manufacturer's instructions, the total RNA was isolated from tissues and cells using Trizol (Invitrogen, USA). RNA concentration was measured using NanoDrop (Thermo Scientific). For CAPN2, RNA was reverse-transcribed into cDNAs using a PrimeScriptOne-Step RT-PCR Kit (Takara, Dalian, China) in accordance with the manufacturer's instructions. The primers were CAPN2 (5′-GTTCTGGCAATACGGCGAGT-3′, forward; 5′-CTTCGGCTGAATGCACAAAGA-3′, reverse); *β*-actin (5′-ACTGGAACGGTGAAGGTGAC-3′, forward; 5′-AGAGAAGTGGGGTGGCTTTT-3′, reverse). The qRT-PCR program was as follows: 50°C for 2 minutes, 95°C for 5 minutes, 40 cycles of 95°C for 15 seconds, and 60°C for 60 seconds. The reactions were performed and analyzed using an Applied Biosystems StepOne Plus Real-Time PCR System (Applied Biosystems, USA). All reactions were run in triplicate and normalized by the internal control products of *β*-actin.

### 2.3. Transfection

CAPN2 siRNA construct was designed and synthesized by GenePharma (Shanghai, China). The sequences of the small interfering RNA (siRNA) targeting CAPN2 (si-CAPN2) were as follows: sense, 5′-GCGAGGACAUGCACACCAUTT-3′; antisense, 5′-AUGGUGUGCAUGUCCUCGCTT-3′. DU145 and PC3 cells were cultured in 6-well plates at 60–70% confluence on the day before transfection. According to the manufacturer's instructions, cell transfection with the siRNA was performed with Lipofectamine 2000 (Invitrogen).

### 2.4. Cell Proliferation Assay

Forty-eight hours after transfection, cells were seeded into 96-well plates at a density of 2,000 cells/well and cultured for 24, 48, 72, and 96 hours. Cell Counting Kit-8 (CCK-8; Dojindo Molecular Technologies, Japan) was used to determine cell proliferation according to the manufacturer's protocol. Absorbance was detected at the wavelength of 450 nm. Three wells were measured for cell viability in each treatment group.

### 2.5. Cell Cycle Analysis

The cell cycle distribution was analyzed by flow cytometry (Becton Dickinson). Forty-eight hours after transfection, cells were harvested, washed twice with ice-cold phosphate-buffered saline, and fixed with 70% ethanol at −20°C overnight. Then, cells were cultured in 50 mg/mL propidium iodide and 1 mg/mL RNase for 30 min at room temperature. Last, the treated cells were analyzed. At least 100,000 cells were acquired for each sample. The experiments were performed in triplicate.

### 2.6. Protein Isolation and Western Blot

CRPC cell lines and Pca tissues were lysed using radio immunoprecipitation assay buffer (Keygene, Nanjing, China) supplemented with protease inhibitors at 4°C for 30 min. The protein samples were electrophoresed in 10% sodium dodecyl sulfate polyacrylamide gel electrophoresis, transferred onto a polyvinylidene fluoride membrane (Millipore), and then blocked for 1 hour with 5% nonfat milk at room temperature. The membranes were incubated overnight at 4°C with primary antibodies for CAPN2 (Abcam, UK), MMP9, AKT, p-AKT, mTOR, p-mTOR (Cell Signaling Technology, USA), MMP2 (Sigma, Sweden), and GAPDH (Bioworld Technology, USA). A horseradish peroxidase-conjugated secondary antibody was incubated in the membrane for 1 hr after three washes with Tris-buffered saline and 0.1% Tween. The blots were detected using chemiluminescence detection reagent (Thermo Scientific). Protein levels were determined by normalization to GAPDH.

### 2.7. Immunohistochemistry

Clinical Pca tissues and adjacent nonmalignant tissues were fixed in formalin, embedded in paraffin, and cut in 5*μ*m thick consecutive sections. After deparaffinization and antigen recovery (in sodium citrate solution, pH 6.0, 20 min, 98°C), the sections were washed thrice in 0.01 mol/L phosphate-buffered saline (PBS) for 5 min each and blocked for 1 h in 0.01 mol/L PBS containing 0.3% Triton X-100 and 5% BSA. CAPN2 primary antibody (1 : 200) was incubated at 4°C overnight. Next day, the sections were washed with PBS and incubated with PBS containing horseradish peroxidase-conjugated IgG (1 : 500) for 1.5 h at room temperature. Then, the slides were performed by diaminobenzidine reaction. Immunohistochemistry for each sample was observed under microscope and repeated thrice.

### 2.8. Cell Invasion Assay

Cell invasion assays were used to investigate the possible effect on metastasis on CRPC cells according to the manufacturer's instructions. Briefly, 5 × 10^4^ cells in 200 mL of serum-free medium were placed in the upper chamber of the transwell (pore size, 8 mm; BD Bioscience) that was coated with Matrigel (BD Bioscience) in accordance with the manufacturer's protocol. Media containing 20% FBS were added to the lower chamber. Following 2 days of incubation at 37°C, the cells remaining in the upper membrane were removed completely by gentle swabbing. And the lower surface of the membrane was fixed in 95% ethanol and stained with crystal violet. Five random fields were counted. All of the experiments were performed in triplicate.

### 2.9. Cell Migration Assay

The cells transfected with si-CAPN2 or NC were grown to 90% confluency in the medium containing 10% FBS. The cell monolayers were then wounded by scratching with a sterile 200 uL tip. After scratching, cells were incubated in serum-free medium. The wound was captured at 0, 24, and 48 hours under a microscope equipped with a camera.

### 2.10. Statistical Analyses

Results are expressed as mean ± standard deviation (SD). Differences between groups were subjected to Student's *t*-test. *P* < 0.05 was considered statistically significant. All of the statistical calculations were performed using SPSS software (Version 13.0 SPSS).

## 3. Results

### 3.1. CAPN2 Was Highly Expressed in CRPC Cell Lines

**Figure 1:**
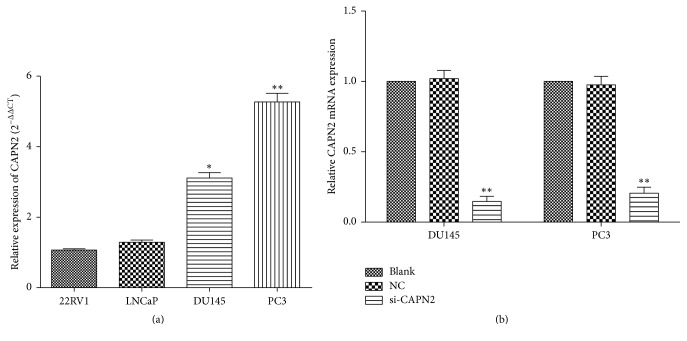
(a) The expression level of CAPN2 in DU145 and PC3 cell lines was relatively higher compared with that in 22RV1 and LNCaP. (b) CAPN2 mRNA expression of the cells transfected with si-CAPN2 was significantly reduced compared with NC or Blank. Data are presented as mean ± SEM. ^*∗*^*P* < 0.05 and ^*∗∗*^*P* < 0.001 by *t*-test.

To investigate the expression of CAPN2 mRNA expression, we used qRT-PCR to show that CAPN2 was significantly upregulated in CRPC cell lines (DU145 and PC3) than other cell lines (*P* < 0.05; Figure 1(a)).

### 3.2. Effects of CAPN2 on Cell Proliferation of Human CRPC Cells

**Figure 2:**
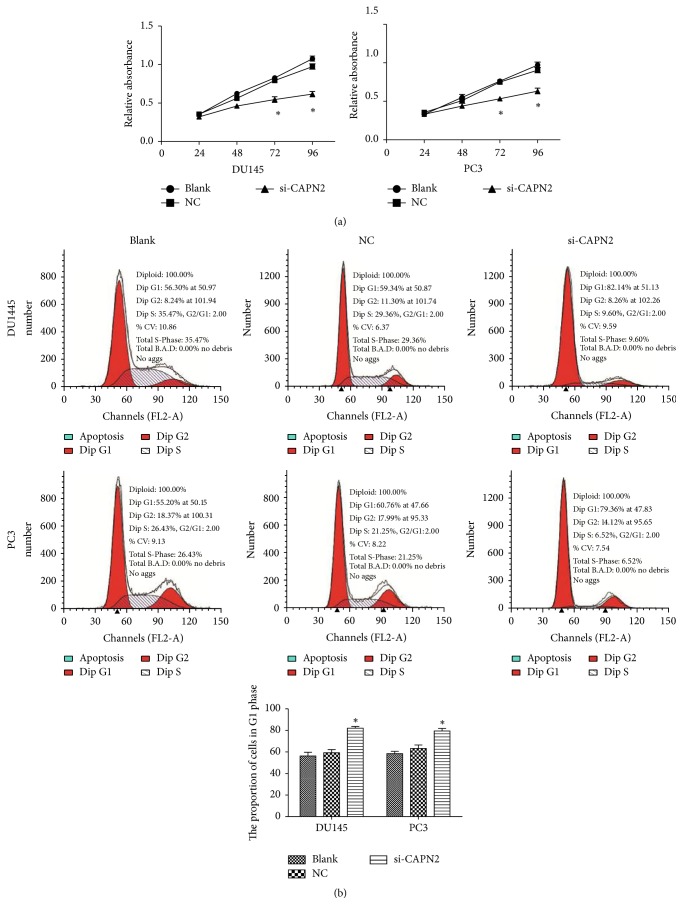
Knockdown of CAPN2 inhibits proliferation in DU145 and PC3 cell lines. (a) CRPC cell proliferation was measured by CCK-8 assay. The results showed that the proliferation of DU145 and PC3 cells was significantly inhibited after being transfected with si-CPAN2. (b) Analysis of cell cycle in CRPC cells indicated that the treated cells were arrested at G1. Data are presented as mean ± SEM. ^*∗*^*P* < 0.05 by *t*-test.

We demonstrated the CAPN2 expression in CRPC cell lines (Figure 1(b)). We further determined the effects of CAPN2 on growth of CRPC cells. DU145 and PC3 cells were transfected with si-CAPN2 or NC (Figure 1(d)). After siRNA transfection for 48 h, we used CCK8 assay to find that downregulation of CAPN2 expression markedly inhibited the growth of CRPC cells at 72 and 96 hours. (*P* < 0.05; Figure 2(a)). Furthermore, we used flow cytometry to analyze cell cycle distribution and to investigate and characterize the effects of CPAN2 on cell growth. The percentages of DU145 and PC3 cells transfected with si-CAPN2 in the G0/G1 phase were higher than that of the NC (*P* < 0.05; Figure 2(b)). These results show that slicing of CAPN2 could inhibit the proliferation of CRPC cells.

### 3.3. CAPN2 Affects Cell Invasion and Migration

**Figure 3:**
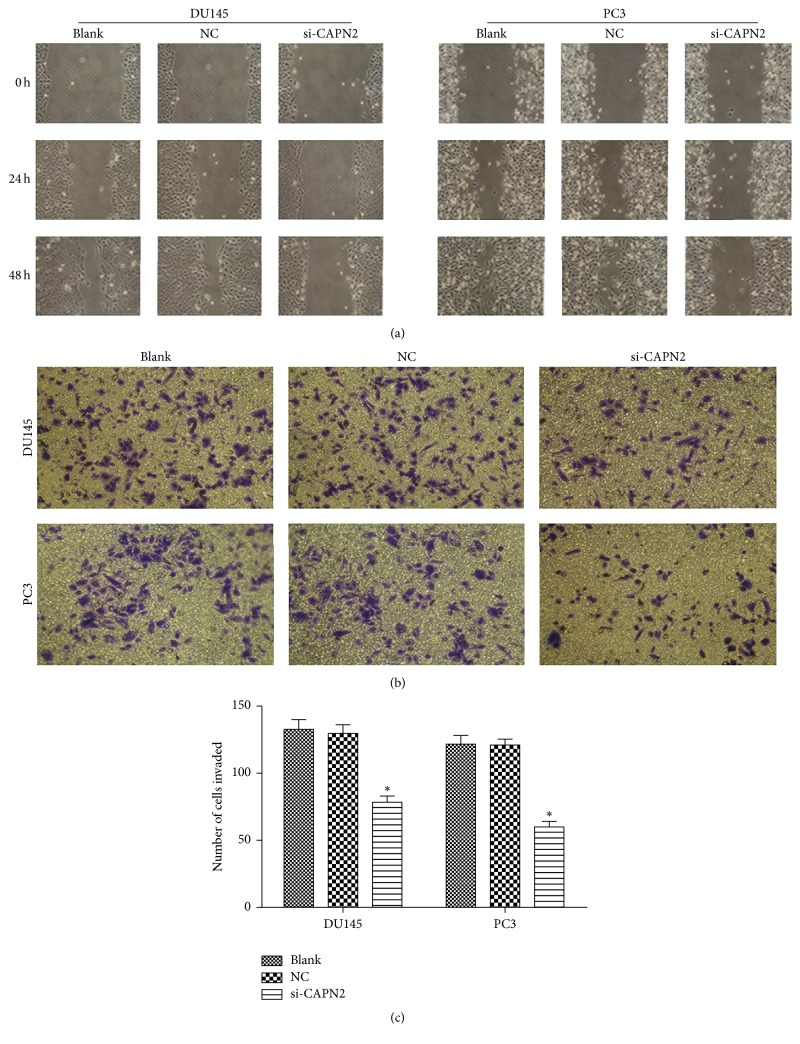
(a) Effect of CAPN2 on wound healing of PC3 and DU145 cells. The invasion of cells in which CAPN2 was downregulated was analyzed by a wound healing assay. (b, c) The decreased CAPN2 expression suppressed the invasion of DU145 and PC3. The invasion of the treated cells was evaluated in an invasion assay using a transwell insert coated with Matrigel. Data are presented as mean ± SEM. ^*∗*^*P* < 0.05 compared with Blank or NC.

After siRNA transfection for 48 h, we used chemotactic cell invasion assay and wound healing to estimate whether CAPN2 was involved in the invasive and metastatic process of CRPC cells. In wound healing, the cells migration was more slower when cells transfected with si-CAPN2 in comparison with NC (Figure 3(a)). Similarly, knockdown of CAPN2 significantly suppressed tumor cells invasion in DU145 and PC3 compared with the NC (Figures 3(b) and 3(c)). All these data strongly show that reducing CAPN2 expression significantly repressed the migratory and invasive abilities of CRPC cells in vitro.

### 3.4. Effects of CAPN2 on Expression of MMP-2 and MMP-9 in CRPC Cells

**Figure 4:**
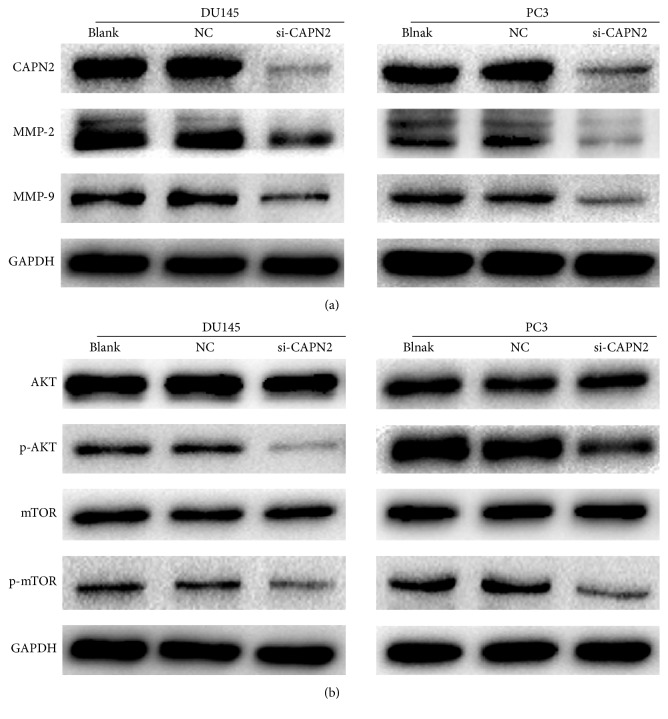
(a) Effects of CAPN2 knockdown on MMP-2/-9 expression levels. (b) Effect of CAPN2 knockdown on AKT/mTOR protein levels. GAPDH was used as a loading control.

As previously described, the MMPs play an important role in degrading ECM, which is required for tumor cell migration and invasion. Western blot analysis revealed a positive correlation between CAPN2 and MMP-2/-9; Western blotting indicated that si-CAPN2 observably decreased the expression of MMP2 and MMP9 when GAPDH served as a loading control (Figure 4(a)). Our results suggest that downregulation of MMP-2/-9 might be involved in reducing migration and invasion of DU145 and PC3 cells after being treated with si-CAPN2.

### 3.5. Effects of CAPN2 on AKT/mTOR Pathways

**Figure 5:**
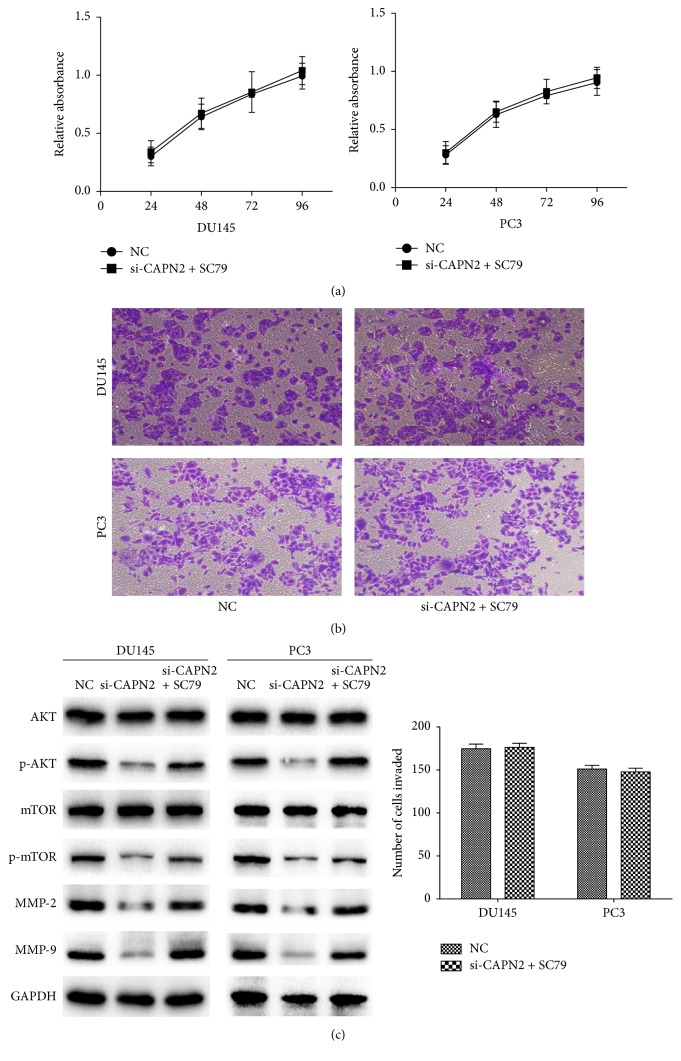
SC79 reversed the inhibitory effects of si-CAPN2 on CRPC cells invasion and proliferation. (a) The proliferation capabilities of DU145 and PC3 cells showed no significant difference between NC and si-CAPN2 + SC79 two groups. (b) SC79 conversed the suppressive effect of si-CAPN2 on CRPC cells invasion abilities. (c) SC79 promoted the protein levels of phosphorylation of AKT/mTOR and MMP-2/9 in si-CAPN2 treated CRPC cells. GAPDH was used as a loading control.

AKT/mTOR pathways were known to play an important role in the proliferation, survival, and motility of tumor cells [15, 16]. Therefore, we used Western blot to investigate the possible effects of CAPN2 on the regulation of AKT/mTOR signaling. CAPN2 protein level was effectively reduced in the treated cells than that in cells treated with control siRNA. The phosphorylation protein levels of AKT and mTOR were significantly repressed after being transfected with si-CAPN2 for 48 hours, while our result shows no impact on the total AKT and mTOR protein (Figure 4(b)). Furthermore, in rescue experiment, SC79 (a phosphorylation activator of AKT) treatment abolished the inhibitory effects of si-CAPN2 on invasion and proliferation of CRPC cells (Figures 5(a) and 5(b)). The expression of phosphorylation-AKT and mTOR also is reversed after being incubated with SC79 (Figure 5(c)). In summary, CAPN2 promoted the invasion and growth capability of CRPC cells by activating AKT/mTOR signaling pathways.

### 3.6. CAPN2 Was Highly Expressed in Pca Tissues

**Figure 6:**
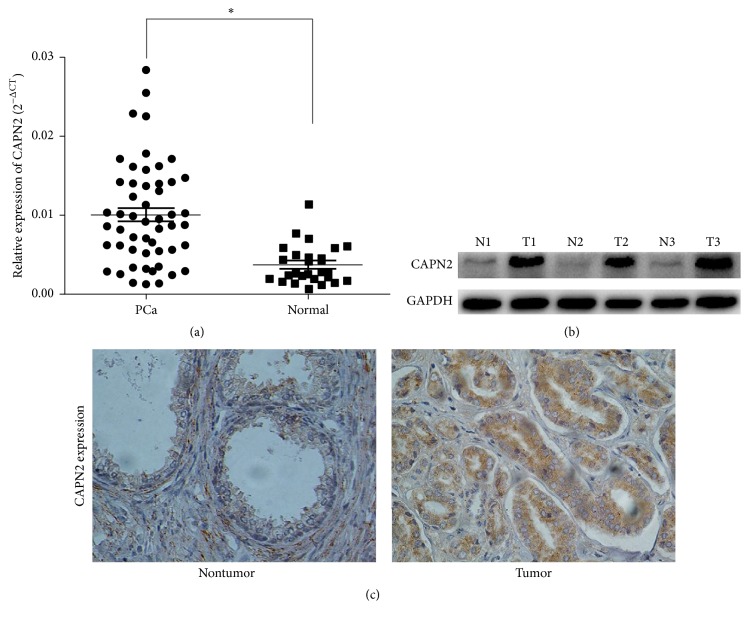
(a) The CAPN2 expression in CRPC tissue and CRPC cells was measured by qRT-PCR and Western blot. (a) CAPN2 expression in CRPC samples was upregulated than that in the nontumor tissues. The median in each triplicate was used to calculate the relative CPAN2 concentration using the comparative 2^−ΔΔCt^ method. (b) CAPN2 protein expression in three matched normal/tumor samples was detected by Western blot analysis. GAPDH was used as an internal control. N and T represent normal and tumor tissues, respectively. (c) Immunohistochemistry staining to examine the CAPN2 expression in Pca tissues and adjacent normal tissues (×400 magnification).

To investigate the expression of CAPN2 mRNA expression, we used qRT-PCR to show that CAPN2 was significantly upregulated in Pca tissues than that in normal control tissues (*P* < 0.05; Figure 6(a)). In addition, Western blot and immunohistochemistry analysis revealed that CAPN2 protein level was higher in tumor samples compared with that in adjacent nonmalignant tissues collected from the same patients (Figures 6(b) and 6(c)). These findings suggest CAPN2 was correlated with tumor progression in prostate cancer.

## 4. Discussion

Accumulated evidences have indicated that CAPN2 play a crucial role in the progression and prognosis of various cancers, including prostate cancers [8–10, 13, 17]. Interestingly, an early study reported that calpain levels were not altered in human prostate tumor [18]. In contrast, an another researcher found that CAPN2 mRNA was significantly upregulated in prostate carcinomas compared with the normal control prostate tissues [14]. In the present study, we detected the mRNA expression of CAPN2 in CRPC cell lines (DU145 and PC3) using qRT-PCR analysis. But the underlying mechanism on CRPC remains to be poorly understood. Therefore, our study aims to show the possible effects of CANP2 on CRPC cells and provide new insights into the progression of CRPC.

CRPC progression was a multiple step, among which was the capacity to reduce the extracellular matrix that stands for a crucial step for tumor invasion and metastasis [19]. The matrix metalloproteinases (MMPs) were known to break down ECM. Numerous studies providing the important roles of MMPs in CRPC metastasis mechanisms have been reported: MMP-2 and MMP-9 protein have been associated with aggressive CRPC and acted as significant prognostic factors in human prostate cancer [20–22]; downregulation of MMP2-/-9 protein levels could reduce the migration and invasion of the treated CRPC cells [23–25]. Previous results have shown that CAPN2 might affect the invasive and metastatic capability of tumor cells in HCC by attenuating MMPs protein levels [10]. Therefore, our study firstly showed the relationship between CAPN2 expression and MMPs in prostate cancer cells. We used mRNA silencing techniques to reduce CAPN2 expression, and our findings indicate that si-CAPN2 suppress the invasive ability and wound healing capacity. Furthermore, knockdown of CAPN2 expression in vitro also showed a significant reduction in MMP2 and MMP9 protein expression, suggesting a strong association of CAPN2 and MMPs.

MMP activity was mediated and controlled by its relevant gene expression and enzymatic reaction. Several evidences have indicated that AKT and its downstream factor mTOR could have effects on MMP-2 and MMP-9 expression [26–28]. The RAS/MAPK/ERK and AKT/mTOR signal pathway has been verified to play critical roles in the development and progression of human carcinomas [26, 29, 30]. Furthermore, the treatment of cancer cells with calpain inhibitors reduced the levels of truncated retinoid X receptor-*α*, which could act to promote cancer cell proliferation and survival through AKT activation [31]. Therefore, we further determined the possible effects of CAPN2 on AKT/mTOR signal pathway. In our study, the results showed that a low expression of CAPN2 expression could significantly repress the phosphorylation protein level of AKT, as well as mTOR, while no significant difference was found in the total AKT and mTOR protein. In addition, CAPN2 also affected the treated cells growth, by arresting in G1 phase of the cell cycle. All these results suggest that downregulation of CAPN2 might suppress CRPC cell proliferation and invasion by reducing MMP-2/-9 through AKT/mTOR pathway.

Furthermore, we observed that CAPN2 mRNA expression was highly regulated in Pca tissues than that in the nonmalignant samples. Analogously, in comparison with the adjacent normal tissues, CPAN2 protein level was significantly upregulated in the tumor tissues by Western blot and immunohistochemistry analysis. It indicated that CAPN2 might act as oncogenic biomarkers and promote prostate cancer progression.

In conclusion, our data indicated that silencing CAPN2 expression could inhibit cell growth, migratory, and invasion ability of CRPC cells by MMP2 and MMP9 enzyme activities. And, we also found that AKT/mTOR pathway was involved in the effect of CAPN2 on the transfected cells. We believe that CAPN2 may provide a new therapeutic strategy for treating prostate cancer patients.

## Figures and Tables

**Table 1:** Patients characteristics.

Characteristic	Pca (*n* = 52)
Age (years)	
Median (range)	67 (54–79)
T stage	
T1	2
T2	30
T3	16
T4	4
N stage	
N0	50
N1	2
M stage	
M0	50
M1	1
Gleason score	
<7	12
=7	28
>7	12
